# Correction: Bertelli et al. Is Less Always More? A Prospective Two-Centre Study Addressing Clinical Outcomes in Leadless versus Transvenous Single-Chamber Pacemaker Recipients. *J. Clin. Med.* 2022, *11*, 6071

**DOI:** 10.3390/jcm12062311

**Published:** 2023-03-16

**Authors:** Michele Bertelli, Sebastiano Toniolo, Matteo Ziacchi, Alessio Gasperetti, Marco Schiavone, Roberto Arosio, Claudio Capobianco, Gianfranco Mitacchione, Giovanni Statuto, Andrea Angeletti, Cristian Martignani, Igor Diemberger, Giovanni Battista Forleo, Mauro Biffi

**Affiliations:** 1 IRCCS Azienda Ospedaliero, Universitaria di Bologna, 40122 Bologna, Italy; 2 Unità Operativa di Cardiologia, ASST-Fatebenefratelli-Sacco, Ospedale Luigi Sacco University, 20157 Milano, Italy

The authors wish to make the following corrections to this paper [1]. 

On page 6 of the published paper, the authors would like to correct Figure 2.

## 1. Error in Figure 2

In the original publication, there was a mistake in Figure 2 as published. Albeit correctly labelled, the leadless and transvenous Kaplan–Meier curves had different colours in the all-cause and cardiovascular mortality graphs, which may cause confusion. The corrected Figure 2 appears below. 

**Figure 2:**
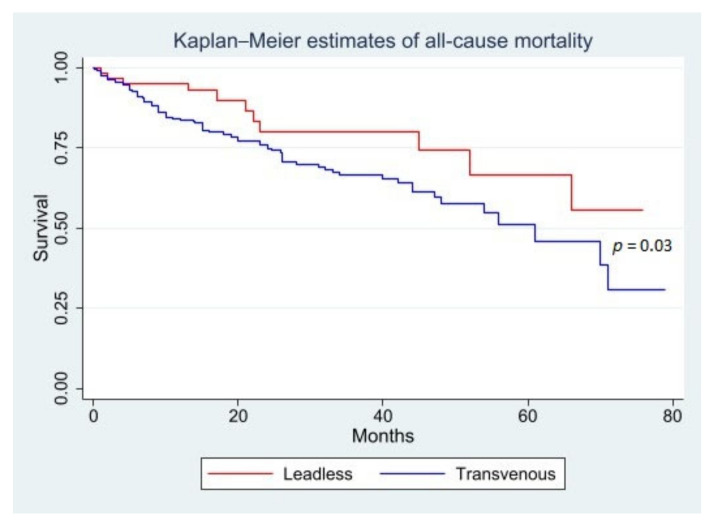

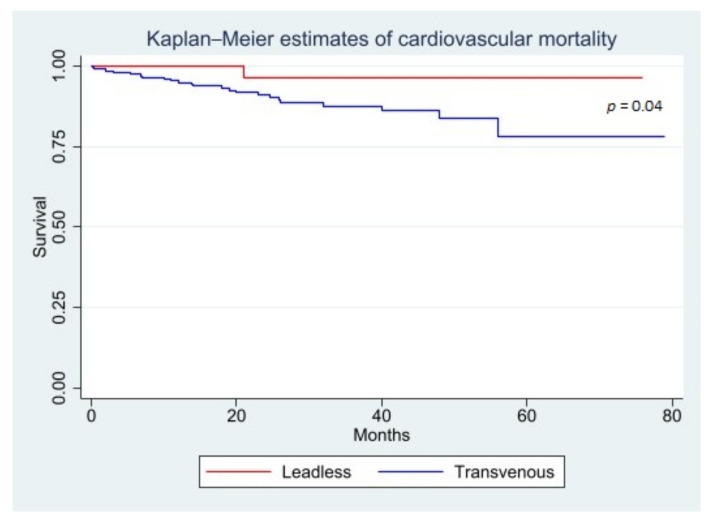
Kaplan–Meier estimates of all-cause and cardiovascular mortality in LL and TV PM recipients over the follow-up period.

On page 7 of the published paper, the authors would like to correct Table 3:

## 2. Error in Table 3

In the original publication, there was a mistake in Table 3 as published. The corrected Table 3 appears below.

**Table 3:** Multivariate analysis of all-cause mortality in LL and TV cohorts and on the entire population.

Multivariate Analysis of All-Cause Mortality in LL vs. TV				
Variable	Leadless (n = 72)		Transvenous (n = 272)	
	Hazard Ratio [95% CI]	*p* Value	Hazard Ratio [95% CI]	*p* Value
Age	1.019 [0.909–1.142]	0.74	1.073 [1.026–1.122]	0.002
Female sex	1.381 [0.227–8.394]	0.73	1.507 [0.881–2.576]	0.13
Diabetes mellitus	1.352 [0.1666–11.037]	0.78	1.864 [1.020–3.406]	0.05
Chronic kidney disease *	0.564 [0.078–4.069]	0.57	1.774 [0.922–3.413]	0.09
Ischaemic heart disease	2.109 [0.421–10.550]	0.36	1.156 [0.666–2.005]	0.61
Left ventricular ejection fraction	0.958 [0.878–1.045]	0.33	1.000 [0.974–1.027]	1.00
Ventricular stimulation percentage	5.856 [0.994–34.484]	0.05	1.128 [0.655–1.943]	0.66
**Multivariate Analysis of All-Cause Mortality in Both Cohorts**				
**Variable**	**Hazard Ratio [95% CI]**		***p* Value**	
Leadless vs. transvenous	0.929 [0.422–2.043]		0.85	
Age	1.071 [1.027–1.117]		0.001	
Female sex	1.473 [0.888–2.444]		0.13	
Diabetes mellitus	1.617 [0.911–2.869]		0.10	
Chronic kidney disease *	1.704 [0.953–3.047]		0.07	
Ischaemic heart disease	1.226 [0.741–2.031]		0.43	
Left ventricular ejection fraction	0.995 [0.971–1.019]		0.67	
Percentage ventricular stimulation	1.347 [0.805–2.253]		0.26	

* eGFR < 60 mL/min/1.73 m^2^.

On page 6 of the published paper, the authors would like to correct the last sentence of Paragraph 3.5 of the Results section:

## 3. Text Correction in Results Section Paragraph 3.5

There was an error in the original publication. The last sentence of Paragraph 3.5 reads: “On multivariate analysis of mortality in TV and LL cohorts, only age in the TV group and ventricular stimulation percentage in the LL group appeared to significantly impact mortality, while on analysis of the entire population, only age displayed a significant association with mortality (Table 3)”; however, no significant impact of ventricular stimulation percentage in the LL group was demonstrated in the analysis. A correction has thus been made to Results, Paragraph 3.5 as follows: “On multivariate analysis of mortality in TV and LL cohorts, only age in the TV group appeared to significantly impact mortality. Similarly, on analysis of the entire population only age displayed a significant association with mortality (Table 3).”

On page 3 of the published paper, the authors would like to correct the third paragraph of the Materials and Methods section:

## 4. Text Correction in Materials and Methods Section

There was an error in the original publication: the symbol “≤” is missing from the second sentence of the third paragraph of the Materials and Methods section. A correction has thus been made to the third paragraph of Materials and Methods, as follows: 

“Specifically, vitamin K antagonists were tapered to the INR range of 2–2.5 for the week following device implantation in patients with CHA2DS2-VASc score ≤ 3.”

Lastly, on pages 3–6 of the published paper, the authors would like to make the following corrections to the Results section:

## 5. Text Correction in Results Section

In the Results section (pages 3–6), the spelled out numbers have been changed to numerals for clarity purposes.

The authors state that the scientific conclusions are unaffected. This correction was approved by the Academic Editor. The original publication has also been updated.
