# Supplementary material for: Is Less Always More? A Prospective Two-Centre Study Addressing Clinical Outcomes in Leadless versus Transvenous Single-Chamber Pacemaker Recipients
Source: J Clin Med. 2022 Oct 14;11(20):6071. doi: 10.3390/jcm11206071 (PMC9604678; doi:10.3390/jcm11206071)
Supplement: Supplementary file 1 [file jcm-11-06071-s001.zip › jcm-1958668-supplementary.pdf]

Supplementary material legend:

Videos documenting the single episode of leadless VVIR pacemaker dislodgement and its management.

- Supplementary material 1: Leadless pacemaker stability testing.
- Supplementary material 2: Dislodgement below the tricuspid valve.
- Supplementary material 3: Pacemaker dislodged into right atrium.
- Supplementary material 4: Retrieval of leadless device.
